# Noninvasive Urine-Based Tests to Diagnose or Detect Recurrence of Bladder Cancer

**DOI:** 10.3390/cancers13071650

**Published:** 2021-04-01

**Authors:** Marine Charpentier, Charly Gutierrez, Thierry Guillaudeux, Grégory Verhoest, Rémy Pedeux

**Affiliations:** 1 COSS (Chemistry Oncogenesis Stress Signaling)—UMR_S 1242, University of Rennes, INSERM, CLCC Eugène Marquis, F-35000 Rennes, France; marine.charpentier@univ-rennes1.fr (M.C.); charly.gutierrez@univ-rennes1.fr (C.G.); thierry.guillaudeux@univ-rennes1.fr (T.G.); 2 Department of Urology, CHU RENNES, Rue Henri le Guilloux, 35033 Rennes, France; Gregory.Verhoest@chu-rennes.fr

**Keywords:** bladder cancer, diagnostic, non-invasive, liquid biopsy, cytology, urine

## Abstract

**Simple Summary:**

Bladder cancer (BC) is the tenth most common cancer worldwide, with approximatively 550,000 new cases and 200,000 deaths in 2018. BC is divided into two subgroups: non-muscle invasive bladder cancer, an early stage of the cancer, and muscle invasive bladder cancer, which is more aggressive. The crucial issue today is to be able to detect BC easily and early, with high sensitivity and specificity, in order to treat it sooner, using less invasive methods. Over the past decade, progress has been made to improve detection methods using novel urinary biomarkers. In this review, we discuss the present and future of noninvasive urine tests to diagnose or detect the recurrence of bladder cancer.

**Abstract:**

Liquid biopsies are increasingly used for the diagnosis and follow-up of cancer patients. Urine is a body fluid that can be used to detect cancers and others diseases. It is noninvasive and easy to collect. To detect Bladder Cancer (BC), cytology is the first assay used. It is an effective way to detect high grade BC but has a high rate of equivocal results, especially for low grade BC. Furthermore, cystoscopy is used to confirm cytology results and to determine cancer status. Cystoscopy is also effective but highly invasive, and not well accepted by patients, especially for BC follow-up. In this review we survey the numerous assays recently developed in order to diagnose BC at an early stage, and to facilitate the follow-up of patients. We discuss their effectiveness, ease of use, and applications. Finally, we discuss assays that, in the future, could improve the diagnosis and management of BC patients.

## 1. Introduction

**Figure 1:**
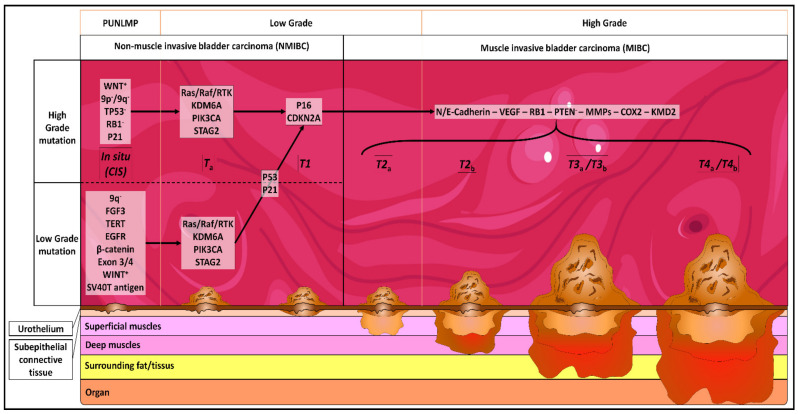
Genetical and physiological evolution of bladder cancer (BC). CIS: carcinoma in situ: “flat tumor”; T*_a_*: noninvasive papillary carcinoma; T1: tumor invades subepithelial connective tissue; T2*_a_*: tumor invades superficial muscularis propria (inner half); T2*_b_*: tumor invades deep muscularis propria (outer half); T3*_a_*: tumor invades surrounding fat/tissues microscopically; T3*_b_*: tumor invades surrounding fat/tissues macroscopically; T4*_a_*: tumor invades prostatic stroma, uterus, vagina; T4*_b_*: tumor invades pelvic wall, abdominal wall. PUNLMP = papillary urothelial neoplasm of low malignant potential.

Bladder cancer (BC) is the tenth most common cancer worldwide, with approximatively 550,000 new cases and 200,000 deaths in 2018. The incidence is 9.6 and 2.4 per 100,000 in males and females, respectively. In France, 12,000 new cases were diagnosed in 2012, and it is the seventh most common cancer [1]. It is the most expensive cancer to treat in Europe [2]. Seventy to eighty five percent of BCs are urothelial carcinoma (UC), commonly termed non-muscle invasive bladder cancer (NMIBC) [3]. Squamous, adenocarcinoma, and small cell carcinoma are less common, and are associated with an advanced stage and higher mortality than NMIBC. NMIBC is an early stage of cancer and is classified as stage Ta to T1; it is only found in the first layer (urothelium) and the second layer (*lamina propria*) of the bladder (Figure 1). Muscle Invasive Bladder Cancers (MIBC) are classified as T2 to T4, and are only found in the third layer of the bladder (*muscularis propria*) (Figure 1). The staging of BC is determined by the TNM system [4].

The best-known risk factor for BC development is tobacco smoking. Smoking is responsible for approximately two-thirds of BCs in men and one-third in women [5]. The second most common risk is exposure to specific chemical products. These products are especially present in paint, plastic, printing, textile, or rubber industries (e.g., aromatic amines, polycyclic aromatic hydrocarbons). The five-year survival rate for low grade NMIBC is 96%, dropping to 35% for MBIC and 5% for metastatic MBIC. With current diagnostic techniques, only 10% of low grade NMIBC are detected, due to the difficulty of differentiating low grade cancerous cells from healthy cells. In recent years, many biomarkers for detecting, in particular, low-grade BC have been described and used to develop new diagnostic tests (Table 1).

**Figure 2:**
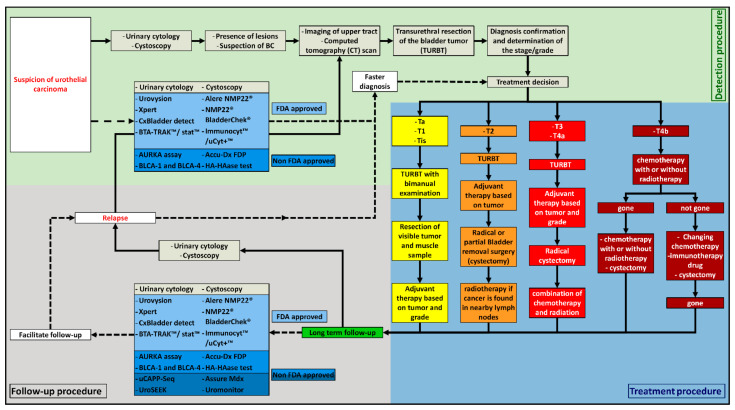
The different ways of detection, diagnosis, treatment, and follow-up of BC, depending on the clinical situation. **――――:** Classical routine used by the clinicians nowadays; **− − − − −**: new methods that can be used by the clinicians nowadays.

The first step to detect BC is the presence of a painless visible hematuria [5], the most common symptom in BC, followed by irritative urinary symptoms. These symptoms could also be due to other diseases, such as renal cancer, prostate cancer, interstitial cystitis, renal calculi, benign prostatic hyperplasia, and trauma [6]. Different tests can be used at this point in order to find out the cause of these symptoms. In the case of BC suspicion, several tests exist (Figure 2). One of them is urine cytology. It consists of a microscopic observation of the cells present in the urine of the patient after Papanicolaou staining. The samples are analyzed by the pathologist according to the Paris System guidelines [7]. Urine cytology is an easy assay to perform, and it has a sensitivity of 37% (95% CI 35–39%) and a specificity of 95% (95% CI 94–95%). It has good results for the detection of high-grade tumors. Moreover, it is a low-cost method. However, the real issue with this test is its low sensitivity for low-grade lesions and its high rate of equivocal results [8]. Therefore, it is always used in combination with cystoscopy to confirm the diagnostic.

Cystoscopy is the gold standard for the detection of BC. It gives information about the number, localization, aspect and size of the tumor(s). It is systematically performed when there is a suspicion of BC with urine cytology [9]. This method has a sound sensitivity (68.3 to 100%) but can give false negative result (due to operator error or the difficulty of finding the tumor because of its small size at the carcinoma in situ stage). It also has a good specificity (57 to 97%) [10]. Cystoscopy is an endoscopic procedure used to look inside the bladder. It is an invasive assay and causes patient discomfort, possible urinary tract infection, and anxiety [10,11,12,13].

A disadvantage of these two tests is their low efficiency for detecting BC at an early stage. Indeed, the detection of early stage BC is a real issue nowadays. Early detection gives the patients a greater probability of being cured from BC and is cheaper to treat. Indeed, BC is an expensive cancer to treat and to survey in comparison with other cancers. Its cost fluctuates between USD 47,500 for distant BC to USD 14,300 during the 1st year after diagnosis, and can reach more than USD 172,000 for a long-term survivor [14]. Over the past years, many new tests have been developed in order to detect BC earlier than with current cytology and cystoscopy, and to improve the specificity of BC diagnosis. The aim of this review is to present BC diagnostic tests that are available and are being developed at the moment. The specificity and sensitivity of each test will be compared to cytology and cystoscopy results, and every positive or negative aspect will be highlighted to have a clear vision of what their use could bring to the field (the summarized information has been compiled in Supplementary Table S1).

## 2. Urine Protein-Based Assays

Lots of studies are being conducted to improve the diagnostic accuracy of urinary tests, both to create an alternative to urinary cytology and cystoscopy and to improve patients’ follow-up. Numerous biomarkers have been found and some of these underwent clinical validation and approval. Urine has lots of advantages for biomarker detection. It is noninvasive, easy to handle, and more than one biomarker can be investigated at the same time. Indeed, lots of proteins or RNAs present in the urine already serve as markers of cellular dysfunctionality [15,16].

### 2.1. FDA-Approved

#### 2.1.1. BTA-TRAK™ and BTA-STAT™

BTA-TRAK™ and BTA-STAT™ are dosages of the Bladder tumor antigen (BTA), which is the human complement factor H-related protein (hCFHrp). hCFHrp plays a major role in alternative pathway regulator factor H. This protein has played a role in the carcinogenesis by giving a selective growth advantage and an escape to the host immune system [17,18]. BTA-TRAK is a urine ELISA test and BTA-STAT™ is a quantitative point-of-care test. It has been shown that BTA-TRAK and BTA-STAT™ sensitivity are better than cytology (57–83% vs. 37% respectively) [19,20,21]. However, their specificity is lower than urinary cytology due to false-positives (51–75% vs. 99%) [20], in particular, nontumor urinary tract diseases [22,23]. That is the reason why these tests are only used for patient follow-up in association with cytology [21]. They can also be detected in urine.

#### 2.1.2. NMP22 (Nuclear Matrix Protein 22) Protein Test

Nuclear matrix protein 22 (NMP22) has an important role in the regulation of mitosis by regulating the distribution of chromatin to daughter cells, and is found in the nuclear matrix of all cell types [24]. NMP22 is highly expressed in bladder tumor cells and released from the nucleus after they die; it can be detected in the urine [25,26,27]. Two tests are available for the dosage of NMP22, an ELISA test (Alere NMP22^®^) or a point-of-care test (NMP22 BladderChek^®^). The sensitivity of the Alere NMP22^®^ test and NMP22 BladderChek^®^ test are higher than cytology alone (68% and 65%, respectively, vs. 37% for the cytology [28]. The grade of the tumor plays an important role in the specificity of the test [29,30]. However, the specificity is still lower than cytology—79% for Alere NMP22^®^ vs 95% for cytology [28]. Unfortunately, the constitutive presence of NMP22 in urothelial cells can induce a false-positive and impair diagnostic specificity [21,22,30]. NMP22 level is not used for the first diagnostic but it helps for the follow up, since it has shown a higher sensitivity than cytology for the low-risk group [31]. Finally, NMP22 BladderChek^®^ is a test which is easy to perform; it gives results in 30 min, in contrast to Alere NMP22^®^, which needs to be performed in the laboratory.2.1.3. Immunocyt^TM^/uCyt+^TM^.

ImmunoCyt/uCyt+ is an immunocytochemical test targeting two antigens and was developed by Fradet and Lockhard in 1997. The first antigen is a superficial bladder cancer-associated sialylated epitope expressed on a heterogeneous group of glycoproteins in the membrane; more precisely, a glycoform of the carcinoembryonic antigen (CEA). This CEA glycoform is a 200 kDa membrane glycoprotein anchored to the membrane via a glycosylphosphatidylinositol link and is highly glycosylated. This glycoprotein is observed in tumor cells only, and preferentially in superficial bladder tumors [32,33,34]. This antigen is targeted by the 19A211 antibody [33]. The second antigen is a secreted high-molecular-mass mucin-like, called mucin antigen of the urinary bladder (MAUB) [35]. It is secreted by mucosae and some exocrine glands [34,35,36,37]. This MAUB is targeted by M344 and LDQ1053 antibodies.

Studies have shown that ImmunoCyt/uCyt+ alone has a sensitivity ranging from 38.5% to 92.1% across all grades and risk categories, which is higher than cytology (from 23% to 84.6%). However, the specificity is better for cytology than for ImmunoCyt/uCyt+, with a range from 62% to 84.2% vs. 79.7% to 99.4% for cytology. Nevertheless, it has been shown that when cytology tests and ImmunoCyt/uCyt+ are used together, sensitivity improves by at least 15%, ranging between 53.8% and 94.1% [38]. Moreover, the sensitivity for low-grade tumor increased from 8.3%, for cytology alone, to 79.3% with a combination of both diagnostics [39]. They also showed that the sensitivity for high-grade was improved, with a sensitivity by 75.3%, for cytology alone, and 98.9% for the combination. Unfortunately, the specificity of the combination is still lower than cytology alone (ranging from 61% to 80.7%) [40,41] but better than ImmunoCyt/uCyt+ alone. In conclusion, this test is useful for improving overall sensitivity when combined with cytology for all grades of BC.

### 2.2. Non-FDA-Approved

#### 2.2.1. Cytokeratins

The first potential role of cytokeratins as tumor markers for epithelial cancer cells has been shown by Björklund B. and Björklund V, in 1957, with the tissue polypeptide antigen (TPA) [42]. TPA is present in the proteolytic fragments of CKs 8, 18 and 19; these fragments are released into body fluids, such as urine and serum, as a sign of cell death [43]. It has been shown that the concentration of the antigen is higher in patients with tumors [44]. Since the discovery of the high level of CKs in BC, several commercially available tumor marker tests have been developed, such as the TPA, tissue polypeptide-specific antigen (TPS) for CKs 8 and 18, tissue polypeptide cytokeratin antigen (TPACYK), and the cytokeratin fragment of CKs 8 and 19 (CYFRA 21-1) [45]. However, since 2016, a new test to detect cytokeratins has been specifically developed for the diagnosis of BC, the UBC^®^ Rapid Test (Concile GmbH, Freiburg, Germany). This test is now the most commonly used for the detection of cytokeratins. It is a point-of-care test. Two tests are available and both measure fragments of cytokeratins 8 and 18; one is a quantitative measurement and the other is a qualitative measurement [46,47]. These cytokeratins are soluble in urine and can be detected quantitatively with monoclonal antibodies using an enzyme-linked immunosorbent assay (ELISA) [48]. The qualitative and quantitative UBC^®^ Rapid Test had a better sensitivity than cytology alone (61.3% and 64.5%), but still a lower specificity (77.3% and 81.8%).

However, it has been shown that the combination of the UBC^®^ Rapid Test with cytology results in a higher overall sensitivity (77.4%). In contrast with cytology alone, sensitivity increased from 21.4% to 50% for detecting low-grade tumors, and from 43.8% to 100% for high-grade cancers, albeit with a reduced specificity from 100% to 77.3% [49].

#### 2.2.2. Lewis X Antigen

Carbohydrate antigen 3-fucosyl-N-acetyllactosamine, also known as Lewis X, is a blood group antigen belonging to the Lewis system. It was first described by Mourant [50]. It is localized on the surface of cells, such as granulocytes, kidney tubules, and gastrointestinal epithelia [51]. This antigen is normally absent from the urothelial cells in adults, but is expressed by more than 90% of papilloma and transitional cell carcinomas, irrespective of the tumor grade or stage of the BC [52]. This test works with a monoclonal anti-Lewis X antibody (P12) directed against the Lewis X determinant [53]. Only old studies have been performed with the use of this marker, and showed a median sensitivity of 75% and a median specificity of 85% [52,54]. Due to the small number of studies, it is still unknown if this biomarker is efficient.

#### 2.2.3. Survivin

Survivin, also known as baculovirus IAP repeat–containing protein 5 (BIRC5) and apoptosis inhibitor 4 (API4), is a member of the inhibitor of apoptosis protein (IAP) family [55]. Survivin participates in the suppression of apoptosis and is a regulator of the cell division [56,57,58]. Survivin is normally expressed during embryonic and fetal development. It is completely undetectable in normal adult tissue, but a vast majority of tumors express Survivin mRNA and protein at high levels. It can be found inside (cytoplasm, nucleus, and mitochondria) or outside (extracellular space through vesicles) the cell [55,59,60,61,62].

In BC, Survivin expression has been observed by immunohistochemistry and may negatively impact the recurrence of the disease [63]. Actually, studies have shown that Survivin has a real interest, with a sensitivity of 100% and a specificity of 80% to 90% [64,65], which means that this marker could be a good test to help in the detection of BC.

#### 2.2.4. Hyaluronic Acid–Hyaluronidase Test (HA-HAase Test)

Hyaluronic Acid (HA) is a nonsulfated glycosaminoglycan and a component of the extracellular matrix in solid and fluid tissues [66]. The concentration of HA is elevated in several cancers and it has been shown that levels of HA are three to five-fold higher in bladder tumor tissue extracts when compared with normal bladder extracts [67]. Moreover, the expression of the hyaluronidases (HAase) (the enzymes capable of degrading the HA) is also highly expressed in BC tissue when compared with normal bladder extracts [68]. Furthermore, it is also shown that levels of HAase are correlated with the tumor grade. This increase of HAase plays a role in the invasive potential of BC [69]. The HA-HAase test is comprised of two ELISA-like assays: the HA test (dosing the HA) and the HAase test (dosing the HAase). The HA and HAase assays use a biotinylated HA-binding protein (HABP) and an avidin-biotin system for detection [22,66,70,71]. A recent meta-analyze showed that the sensitivity of the test is 90.8% for all tumors, with a specificity of 82.5% [66,72,73]. These results suggested that HA-HAase are good biomarkers for the diagnosis of BC.

#### 2.2.5. BLCA-1 and BLCA-4

Bladder Cancer–Specific Nuclear Matrix Proteins (BLCA) are proteins only found in BC. It has been shown that these proteins are associated with tumor cell proliferation, survival, and angiogenesis [74,75]. BLCA-4 is one of the most abundant of this family of six proteins (BLCA-1 to -6). They have been detected in the entire bladder, including the tumor and normal adjacent areas, of individuals who have bladder cancer [74,75]. BLCA-1 is also one of the most abundant and has been shown to be significantly highly expressed in patients with BC than in normal individuals; however, with no correlation with tumor grade. For BLCA-1, a preliminary study showed a sensitivity of 80% and specificity of 87%, demonstrating the potential interest of a BLCA-1-based assays in diagnosis and surveillance of patients with BC [76]. BLCA-4 expression does not appear to be affected by tumor grade or various benign urologic disorders [77]. The protein level in urine was tested using ELISA. A pooled analysis estimated a sensitivity of 93% and a specificity of 97% for BC [78,79].

Current studies suggest that BLCA-1 and BCLA-4 are promising markers in BC diagnostics. Nevertheless, more studies are needed to confirm that BCLA-1 and BLCA-4 are good markers for BC detection.

#### 2.2.6. Fibrin–Fibrinogen Degradation Product (FDP), the Accu-Dx FDP

It has been shown that BC produces a high level of vascular endothelium growth factor (VEGF) [80]. VEGF increases the permeability of the tumor vessels, which leads to leakage of plasma and blood proteins, such as plasminogen and fibrinogen, and coagulation factors into the extracellular space. Coagulation factors rapidly transform fibrinogen into fibrin, which is secondarily degraded by plasmin into FDP [81]. Increased urine fibrin/FDP levels have been shown with the presence of BC [82]. Since the middle of the 1970s, the identification of FDP in urine has been evaluated as a diagnostic test for BC. The Accu-Dx FDP, a qualitative test, is a rapid immunoassay measuring FDP in the urine in a few minutes [82,83]. Studies have shown a sensitivity ranging from 52% to 83% and a specificity from 68% to 86%. Due to a problem with technical stability during manufacturing, the marketing of the Accu-Dx FDP has been stopped [84].

### 2.3. Developing Diagnostics

#### 2.3.1. Tumor-Secreted Extracellular Vesicles (EVs)

Recently, a small study identified 4 proteins as potential biomarker candidate proteins in EVs secreted by BC cell lines using mass spectrometry, which could be useful for diagnostics (HEXB, S100SA4, SND1 and EHD4) [85]. Moreover, using mass spectrometry, another study found 2 proteins to be possible diagnostic urinary EV biomarkers, α-1-antitrypsin and H2B1K [86]. The authors showed that, when these proteins are combined for the detection of BC, they have a sensitivity of 62.7% and a specificity of 87.59%. Another protein, the periostin present in urinary EVs, has been shown to be significantly higher in urinary EVs from patients with BC than healthy participants [87]. Periostin plays an important role in the invasion in BC. However, more data still need to be collected to evaluate the real efficiency of EV proteins as biomarkers, as not many studies have been performed, covering a small number of patient samples.

#### 2.3.2. Urinary Midkine Protein

Midkine protein (MK), also known as neurite growth-promoting factor 2 (NEGF2), is a heparin-binding growth factor involved in several pathways, such as growth factor activities in cellular proliferation, survival, and migration. In addition to the growth factor activities, MK also plays a role in fibrinolysis, blood pressure, host defense, and other processes [88,89]. In adult physiological condition, MK is only detected in kidneys at a very low level [90]. In contrast, MK was demonstrated to be significantly upregulated in various human cancers, such as BC [91]. This observation has been correlated with previous studies, which described that the upregulation of MK is also observed in urine specimens of BC patients [92,93]. It has been shown that the expression of MK is correlated with a poor outcome in patients with invasive BC, with a higher concentration in urine in advanced stages of BC [94]. Unfortunately, a recent study showed that the dosage of the urinary MK is less efficient than a urinary cytology, with a sensitivity of 69.7% vs. 87.6% and a specificity of 77.9% vs. 87.7%. The combination of dosages of the urinary MK with the urinary cytology improved the sensitivity to 93.3% with, as a counterpart, a reduction of the specificity to 66.2% [94]. Thus, urinary MK could potentially be suitable for the identification of patients with a high risk of BC.

#### 2.3.3. Liquid-Based Cytology (LBC) in BC and Quantitative Proteomic Analysis

LBC is an innovative slide-making technique. It has been widely used for several cancers, such as cervical cancer, breast cancer, sputum cytology of lung cancer, and now, in BC. The LBC allows a better quality of the samples by reducing the time to make a sample slide, removing the nonurothelial cells and mucus in the urine, humidifying slides, and decreasing cell degeneration using a preservation solution. It also improves the microscopic analysis of the slides with a better background, a better cell dispersion, and with less atypical cells than in urinary cytology [95]. A recent meta-analysis shows that LBC has no significant sensitivity improvement when compared to a traditional urinary cytology, with a sensitivity of 58% and specificity of 96% [95]. But it was shown that LBC is more efficient in detecting malignant cells in comparison with urinary cytology (37.3% vs. 25.3%) [96]. More studies are needed to evaluate if LBC is a good method to replace classical cytology. Finally, a recent study shows that LBC could be a useful tool to detect new clinical biomarkers [97]. The authors generated the first and largest in-depth quantitative proteomic analysis of BC using LBC. They were able to detect a unique intracellular protein, the neuroblast differentiation-associated protein, AHNAK, with a different expression and localization between tumor and nontumor cells. However, further investigation is required to understand the importance of this protein.

## 3. Urine DNA/RNA Based Assays

Another way to diagnose cancer is to use DNA or RNA released or contained by malignant cells. One of the advantages of using DNA or RNA is that it can be amplified and can be used at early stages. Here, we are presenting tests that have been developed for BC.

### 3.1. Commercialized Urine DNA/RNA Tests

#### 3.1.1. Urovysion

Urovysion is a 4 target multicolor FISH-based test, developed in 2000, to detect BC in urine [98]. The probe set is composed of directly labeled DNA probes targeting pericentromeric regions of chromosomes 3 (CEP3), 7 (CEP7), and 17 (CEP17), as well as the 9p21 locus (LSI 9p21) to quantify the homozygous deletion of the p16 tumor suppressor gene. A 4′-6-diamidino-2-phenylindole stain also allows the evaluation of atypical nuclear features; in fact, polysomic cells tend to have large and irregular nuclei. In their initial paper, Sokova et al., tested Urovysion in 21 BC patients and 9 healthy donors to determine the optimal set of FISH probes, and then in 179 patients, including 93 with BC history and 86 who were healthy, for validation [98]. They found a sensitivity of 84% and a specificity of 92%. In another study, they compared the Xpert assay (see below) to the Urovysion assay on 239 patients with a history of BC, and found a sensitivity of 74% (83% for high grade BC) and a specificity of 80% [99]. Numerous publications have been released since then, mostly about trials to validate Urovysion use as a BC surveillance test. One study, published in 2007, followed 250 patients with BC for 23 months [100]. They compared Urovysion tests to cytology and found that Urovysion is more sensitive at detecting BC recurrence (74% compared to 61%). Another study, published in 2018, assessed the potential benefit of using two Urovysion tests 3 months apart onto specificity and sensitivity of this test for BC recurrence [101]. The authors tested this hypothesis in a cohort of more than 400 patients who had been treated by transurethral resection of bladder tumor for BC and diagnosed within 2 years. If, with the first tests (Urovysion and Cytology), a new tumor was found, the trial ended for them; if nothing was suspicious, then they underwent the same tests 3 months later. By using 2 Urovysion tests, BC recurrence detection was increased from 50% to 72%, and 42% of low-grade BC and 67% of high-grade BC were detected against 0% and 11% using cytology.

Urovysion is a noninvasive test that allows the detection of BC, independent of specific mutations. This test is far more efficient for detecting BC recurrence than cytology. However, one of the problems that can come out using Urovysion is that it can detect other types of cancer, such as kidney cancer, due to leakage of cells in the urine. Urovysion has also a high false-positive rate of 27.6% [101]. Thus, Urovysion may not replace cytology and cystoscopy for the initial detection of BC; the two golden techniques are more efficient. That said, this assay could be used for the follow-up of BC patients. Despite the false-positive rate, the use of Urovysion, instead of cytology, could improve confidence with regard to detecting BC recurrence and diminish cystoscopy frequency, especially if two consecutive Urovysion tests are used. The Urovysion assay has been approved by the FDA in 2001.

#### 3.1.2. Xpert Bladder Cancer Monitor

The Xpert bladder cancer monitor assay analyzes five mRNA targets (ABL1, CRH, IGF2, UPK1B, and ANXA10), frequently overexpressed in BC, using real-time-PCR (Polymerase Chain Reaction) [49,99]. ABL1 serves as a sample adequacy control to ensure the presence of human cells in the urine sample. The Xpert BC monitor is used as a kit. Briefly, cells are captured on a filter, lysed by sonication, and RNA is used for real time PCR. The Xpert bladder cancer monitor has first been compared to cystoscopy and cytology for the follow-up of 140 NMIBC patients [49]. In term of sensitivity, the Xpert assay reached 84% and cytology reached 33%. For specificity, the Xpert assay reached 91% and cytology reached 94%. The combination of both Xpert BC monitor and cytology did not enhance diagnostic performances. This test is more efficient in detecting BC recurrence than cytology. However, it does not seem that the Xpert assay will replace cytology and cystoscopy for the initial detection of BC; the two golden techniques are still more trusted and could cover more cancer types than the Xpert bladder cancer monitor, which focuses on only 5 mRNAs and is not representative of every BC type. However, for the follow-up of patients, Xpert assays could be used as replacements for cytology to improve confidence at detecting BC recurrence and diminish cystoscopy frequency.

#### 3.1.3. CxBladder Detect

The CxBladder detect assay is based on the quantification of 5 mRNA biomarkers found in urine. Four of these biomarkers (IGFBP5, HOXA13, MDK, and CDK1) are associated with growth and propagation of tumor tissue, whereas the fifth biomarker, CXCR2, is a marker of inflammation highly expressed in neutrophils. In this context, CXCR2 enables one to separate patients with BC from patients with inflammation alone [102,103]. In the initial study, 36 patients with no prior history of BC, 39 patients under surveillance for BC recurrence, and 77 patients with nonmalignant diseases were followed [102]. For low grade BC, a sensitivity of 47% was achieved and 100% was achieved for high grade BC. Another study compared the CxBladder assay and cytology [103]. They worked on 485 patients with gross hematuria, but without history of BC, and obtained their voided urine before cystoscopy. They obtained a sensitivity of 82% for the CxBladder assay (97% for high grade BC) against 56% for cytology. CxBladder specificity was 85% and 94% for cytology, showing a higher false-positive rate. Using the different markers, they can distinguish between low- and high-grade BC, with a sensitivity of 91% and a specificity of 90%. CxBladder will not replace cystoscopy for the initial detection of BC, but it could replace cytology because it is more efficient and makes it easier to separate low- and high-grade BC. Despite the false-positive rate, the use of CxBladder in replacement of cytology could improve confidence at detecting BC and diminish cystoscopy frequency [104]. Darling et al. performed a study on 33 patients with 12 urologists, resulting in 396 patient–urologist interactions [105]. All urologists changed their final diagnostics in at least one patient case with the addition of Cxbladder results. The total number of requested invasive procedures was reduced from 425 to 379 (−11%) following disclosure of Cxbladder information. Another study obtained the same results—a reduction in the number of total and invasive procedures [106]. The CxBladder detect assay is FDA approved.

### 3.2. Non-Commercialized Urine DNA/RNA Tests

#### 3.2.1. uCAPP-Seq

uCAPP-Seq is a novel high-throughput sequencing (HTS) method for the detection of urine tumor DNA (utDNA) called utDNA CAPP-Seq (uCAPP-Seq) [107]. DNA contained in urine is purified and sequenced. Around 311 kb of genome are covered, including 460 genes. At first, an analysis of 67 healthy adults and 118 BC patients with different disease stages was performed [107]. The authors found a median of 6 mutations per tested patient; around 70% of mutations found in urine were the same found in the tumor. The two most common mutated regions were TERT (74%) and PLEKHS1 promoters (46%). BC common gene mutations, such as TP53, FGFR3, ERBB2, and RB1, were found, but there was no correlation between the total counted mutations and the stage of the disease. However, there was a correlation between urine DNA concentration and BC risk. As a new diagnostic test, uCAPP-Seq identified 77% of low-grade cancer and 100% of higher stage cancer. For surveillance of BC, cytology was positive for 37.8% of the patients who developed recurrence, while utDNA was positive in 84% of the cases. uCAPP-Seq is more efficient in detecting BC recurrence than cytology; it has a higher rate of detection for low-grade BC than cytology. There is only one publication on uCAPP-Seq, and more work needs to be done to compare and validate this new test to other techniques. It will probably be expensive, but not as expensive as cystoscopy, and costs can be reduced, especially if this technique is used widely. The uCAPP-Seq assay is not yet commercialized.

#### 3.2.2. UroSEEK

The UroSEEK assay enables the detection of specific DNA mutations contained in exfoliated urine cells to diagnose BC and upper tract urothelial carcinomas [108]. This assay targets intragenic mutations in specific regions of the ten following genes (FGFR3, TP53, CDKN2A, ERBB2, HRAS, KRAS, PIK3CA, MET, VHL and MLL), which are frequently mutated in urothelial tumors. It also targets mutations in the TERT promoter and detects aneuploidy. It is based on a singleplex PCR assay to analyze the TERT promoter region and a multiplex PCR assay to analyze the 10 genes regions, followed by sequencing [109]. By adding barcodes to the primers, it could detect mutations in as few as 0.03% of urinary cells. UroSEEK has been tested in three independent cohorts of patients [108]. For the BC early detection, results were compared, when possible, to mutations found in the biopsy of the initial tumor. By testing mutations of only 10 genes, there were false-negative results due to a low number of cancer cells in the urine (62% of the cases) or to the absence of mutations in the tested genes (38% of the cases). By combining the three tests (10 genes mutations, TERT mutations, and aneuploidy), false-positive results were drastically reduced, with only 1 positive result among the 188 healthy patients, reaching a sensitivity of 83%. Among the 395 patients in the BC early detection cohort who did not develop BC during the course of the study, 6.5% scored positive with the UroSEEK test. Despite the false positives, BC was detected 2.3 months before the diagnosis with gold standard techniques (cytology and cystoscopy), and, for 8 cases, more than one year before. By combining a complete UroSEEK test with the golden standard diagnosis test (cytology), a sensitivity of 95% was achieved, a 12% increase over UroSEEK and a 52% increase over cytology and a 93% specificity. For the BC surveillance cohort, a specificity of 80% and sensitivity of 71% were achieved; in combination with cytology, a specificity of 82% was reached. The UroSEEK test was positive 7 months before BC recurrence was diagnosed.

Another study was conducted in 2019 on 527 BC cases, including 373 low-grade and 154 high-grade bladder carcinomas (high grade) treated with transurethral resections or cystectomies [109]. Tumor and not urine-contained cells were analyzed, and white blood cells from healthy patient were used as control. A total of 92% of BC tumors were positive for at least one genetic alteration covered by UroSEEK test, including 70% positive for TERT mutations.

UroSEEK allows false-positive and false-negative diagnostics. False-negative results are mostly BC induced by mutations not detected by the UroSEEK test and, also, because of the low number of cells contained in urine. In fact, for some cancer, no cells were released into urine, at least at the early stage, and they can be diluted with normal cells [110]. UroSEEK should never be used as the only diagnostic test because there are too many false-negative results. However it could be useful for detecting BC recurrence, as it can detect cancerous cells seven months before currently used techniques (cytology and cystoscopy), and mutations covered 90% of BC [109]. Even with a false-positive, it will help to drastically diminish the use of cystoscopy. In order to avoid false-negative results during follow-up, primary tumors could be sequenced to validate if this cancer can be detected by the UroSEEK assay. the UroSEEK assay is not yet commercialized.

#### 3.2.3. Assure Mdx

In the Assure Mdx assay, DNA is extracted from voided urine and analyzed for mutations in FGFR3, TERT, and HRAS, and methylation of OTX1, ONECUT2, and TWIST1 [111]. Assure Mdx was tested on 200 patients, including 97 with BC and 103 with nonmalignant profiles. The authors observed a sensitivity of 93% and a specificity of 86% for BC detection. These first results are promising but more work is needed to conclude on this technique, including comparison to golden standards, at least with cytology. Preliminary results suggest that Assure Mdx could be used for BC follow-up to help reduce cystoscopy and, perhaps, to help for the first diagnostic if sensitivity and specificity are better than with cytology. Assure Mdx is not yet commercialized.

#### 3.2.4. AURKA

The Aurora kinase A (AURKA) gene encodes a key regulator of mitosis and is frequently amplified and/or overexpressed in cancer [112]. The level of AURKA amplification is associated with the level of aneuploidy. AURKA overexpression is associated with poor clinical outcomes due to increased cell cycle progression and development of genomic instability with aneuploidy [113,114]. In this assay, the AURKA gene copy number and DNA ploidy (centromeres for chromosomes 3, 7 and 17) are analyzed by FISH [112]. If three or more AURKA copies are detected, the sample is considered positive for BC. Firstly, 23 BC patients and 7 healthy control (training set) were analyzed, and then 100 BC patients and 148 control subjects (92 healthy individuals and 56 patients with benign disorders) were analyzed [112]. A specificity of 96.6% and a sensitivity of 87% were found. Another study based on 232 patients with BC and 255 control samples, as well as 126 from healthy individuals and 129 from patients with benign urologic disorders, was led. A FISH test for the AURKA gene copy number in urine yielded to a specificity of 80% and a sensitivity of 80% [113]. One of the problems resulting from using AURKA is that, when it is positive, the type of cancer concerned is not clear; it could be kidney cancer or other types of cancer with a leak of cells into urine. AURKA is also a target for the treatment of BC; AURKA tests can only be used prior to any treatment, for initial BC detection. The AURKA based assay is not yet commercialized.

#### 3.2.5. DNA Methylation

There are a multitude of urine DNA methylation-based assays that are developed to detect BC. In fact, gene methylation is different in cancerous cells and can help to distinguish them from healthy cells and, also, from other cancer types or grades. DNA methylation is correlated with poor survival in BC patients [115].

One of the tests allows the quantification of EOMES, HOXA9, POU4F2, TWIST1, VIM, and ZNF154 methylation levels by real-time PCR (MethyLight) [116]. A total of 390 urine samples from 184 patients with low grade BC and 35 healthy patients were studied. For all six markers, independently, a sensitivity between 82% and 89% and a specificity between 94% and 100%, for BC first detection, were obtained. For BC recurrence surveillance, a sensitivity between 88% and 94% and a specificity between 43% and 67% were obtained. It appears that hypermethylation was consistently present in urine samples for a group of healthy patients (*n* =15–31%).

Another test quantifies promoter methylation of 8 genes (ARF, TIMP3, RAR- β2, NID2, CCNA1, AIM1, CALCA and CCND2) by quantitative methylation-specific PCR (QMSP) [117]. A total of 17 nonrecurrent and 19 recurrent BCs were tested to identify optimum combinations, and new markers were found to study and explore. Other prospective confirmatory studies are needed to validate and optimize this assay. A larger cohort is, in fact, needed to obtain specificity and sensitivity rates.

Epicheck is an assay assessing the methylation status of 15 methylation markers [118]. D’Andrea et al. investigated the clinical utility and influence on decision making of the Bladder Epicheck assay for the surveillance of NMIBC using 440 patients [119]. A specificity of 88% was found to detect BC relapse.

Another study investigated voided urine methylation status to identify BC presence and grade [120]. Methylation status of 5 genes (TWIST1, RUNX3, GATA4, NID2 and FOXE1) was studied with a qPCR-based MethylLight assay. Using 211 BC patients (including 180 low grade) and 102 controls, they finally focused on 2 different markers and obtained a sensitivity of 76% and a specificity of 83% for BC detection, and 78% and 61% for grade determination.

A test developed for cervical cancer detection with DNA methylation, GynTect, has been used to detect BC [121]. It usually permits the quantification of DNA methylation on 6 genes (ASTN1, DLX1, ITGA4, RXFP3, SOX17 and ZNF671). By modifying the algorithm, reducing the assay to 4 genes (DLX1, ITGA4, SOX17 and ZNF671), and testing on 30 patients with NMIBC and 30 control subjects, a sensitivity of 60% and a specificity of 96.7% were obtained. By adding others markers known to be modified in BC, this test could be optimized.

As DNA methylation may be dependent on patients’ ethnicities, assays are developed on a specific cohort, such as one on a Chinese patient [122]. A combination of 7 genes (HOXA9, ONECUT2, PCDH17, PENK, TWIST1, VIM and ZNF154) was used to assess its ability to detect BC using a high-resolution melting-curve assay. Encouraging results were obtained on a small cohort, and a larger cohort is scheduled.

DNA methylation quantification is a noninvasive test that enables the detection of BC, independent of specific mutations. One of the problems that may result from using DNA methylation is that, when it is positive, the cancer origin is not defined; it could be kidney cancer or any other type of cancer with cells leaking in the urine. As a minimum number of cells is necessary to detect methylation, 35% of patients were excluded because of an insufficient amount of DNA [116]. Next, a collection of 50 mL of urine or a use of alternative techniques, such as nested PCR, for example, are recommended by the authors. Another limitation could be the treatment used, such as BCG, mitomycin C, etc. Indeed, many of them could alter methylation status and there is a real lack of results in this field. It does not seem that DNA methylation quantification can replace cytology and cystoscopy for the initial detection of BC presently; the two golden techniques are more efficient. But this new technique has better sensitivity than cytology, and could, therefore, replace this technique for recurrence surveillance, allowing for the reduction of cystoscopy frequency.

#### 3.2.6. Uromonitor

The Uromonitor assay allows for the detection of TERT promoter mutations and FGFR3 hotspot mutations in tumor cells exfoliated into urine samples [123]. Cells are filtrated from urine and analyzed with multiplex competitive allele-specific discrimination PCR. The Uromonitor assay has been tested in 185 samples to assess its sensitivity and specificity against cytology/cystoscopy. A sensitivity of 73.5% and a specificity of 93.2% for BC recurrence detection were obtained. These results are comparable to cystoscopy and better than cytology, according to the authors [123]. When used with cystoscopy, they obtained 100% sensitivity and 88.6% specificity. This assay could be completed with the detection of KRAS, as it shows encouraging results. However, 20% of BC does not seem to be detected by the Uromonitor assay, probably due to the fact that not all BCs are reported to have TERT promoter and FRGFR3 mutations. Uromonitor could be used for monitoring BC recurrence, and not BC primary diagnostic, because more than 20% of first BC tumors are not due to TERT promoter or FGFR3 mutations. The use of Uromonitor in the replacement of cytology could improve confidence at detecting BC recurrence and diminish cystoscopy frequency.

## 4. Discussion

Liquid biopsies are increasingly used for the diagnosis and follow-up of cancer patients. Indeed, urine and blood are largely used as liquid biopsies to detect markers, such as circulating cell free DNA, microRNA, circular RNA, non-coding RNA, proteins, cells etc. [124,125,126]. Urine is a body fluid that can be used to detect cancers and others diseases. It is noninvasive and easy to collect. Other types of cancer, completely independent of the genitourinary system, can also be detected using urine, such as breast cancer or Glioblastoma [124]. To detect BC, cytology is the first assay used. It is an effective way to detect high-grade BC and CIS, but with a high rate of equivocal results, especially for low-grade BC. As such, cystoscopy is used to confirm cytology results and to determine cancer status. Cystoscopy is also effective, but invasive, recurrent, and not well accepted by all patients, especially during BC follow-up. As shown in this review, numerous assays were developed during the past few years in order to diagnose BC at an early stage, and to facilitate the follow-up of patients. For BC screening, an assay should be less invasive (noninvasive, if possible) with a high sensitivity, especially for low grade BC, but it is also important to have a high specificity to avoid further unnecessary invasive procedures. Therefore, it should not be solely based on morphology and should be sufficient to detect BC at any stage. Most of the assays described in this review do not fit these criteria, or, at least, are used in combination with cytology and cystoscopy, such as ImmunoCyt/uCyt+^TM^. Some assays are dedicated to one specific stage of BC, such as Assure Mdx and Xpert assays. Some of the tests could not be used for the initial diagnostic, such as NMP22 BladderChek^®^, Urovysion or Cxbladder. This is one of the critical points—the tests do not allow detection of BC at an early stage with high sensitivity.

For diagnosis, an assay’s specificity and sensitivity must be very high. Price should not be an issue, as a low number of persons would undergo it based on an efficient screening assay. None of the assays described above can compete with cystoscopy for definitive diagnosis.

For the follow-up of patients, one of the major issues is the invasiveness of tests and their repetition over time. The recurrence of these invasive tests can lead to urinary tract infections, pain, or discomfort, leading to the patient’s own discontinuation of follow-up care. In addition, lifelong follow-up leads to heavy financial burdens. Therefore, noninvasive assays with a high specificity, high sensitivity, and moderate cost would be greatly beneficial. Many of the assays described here could, or are, used as follow-up assays, especially because some of them have high specificity and sensitivity, and are specific to stages of BC and as easy to perform as BTA assays. Therefore, follow-up assays could be selected according to patient profile and effectiveness. Urovysion assays and UroSEEK assays seem to work for BC recurrence detection.

To date, assays are efficient in the advanced BC stage for the diagnosis and follow-up of patients. Unfortunately, for the initial screening of patients, especially for low-grade cancer patients, assays lack effectiveness. These types of assays are crucial, as they could save lives by detecting BC sooner. They could also considerably reduce overall BC cost. New assays are in development and there are not enough data available yet to describe them in this review, but they could fill some of the gap in the future. Thus, miRNA could be used to develop diagnostic tests for bladder cancer, since they are stable in body fluids and could, therefore, be easily used to develop noninvasive tests. In fact, several miRNA misregulations are associated with bladder cancer development and outcome prediction, such as miRNA-155, 21-miRNA, miRNA-1178, etc. [127,128,129]. An interesting meta-analysis about urine miRNA tests to detect BC was published in 2018 [130]. According to Kutwin et al., a test studying multiple miRNA, and not just one, could compete with cytology tests; however, there is still a need to perform clinical trials to validate this hypothesis. Another possible future test is based on an improved cytology reading thanks to artificial intelligence. A specific urine preparation could allow the visualization of urothelial cells, coupled with an image processing algorithm of urinary cytology slides. This assay, called Visiocyt, is described as sensitive for low-grade BC. Other assays could rely on next generation sequencing (NGS), allowing detection of a low amount of cancer DNA in urine.

## Figures and Tables

**Table 1:** Genes altered in BC and used to develop urine-based diagnostic tests.

Pathway	Altered Genes	Denomination	Activity	Fonction	Status in Bladder Cancer	Alterations Consequences in BC/Remarks	Publications
DNA damage signaling and repair	ERCC2 (XPD)	ERCC Excision Repair 2	Part of transcriptional initiation factor (TFIIH)	Involved in transcription-coupled nucleotide excision repair	Muations leading to inactivation	Point mutation accumulation. Vulnerability to cisplatin chemotherapy.	Kim et al., 2016 Liu et al., 2016
	ATM	Ataxia telangiectasia mutated	Serine/threonine protein kinase	Initiates DNA damage checkpoint activations leading to cell cycle arrest, DNA repair or apoptosis	High expression level or mutations	Mutations accumulation. Promotes proliferation. Radiotherapy resistance.	He et al., 2017
	RB1	Retinoblastoma-associated protein	tumor suppressor	Key cell division regulator	Mutations		Yin et al., 2018
	APOBEC	Apolipoprotein B mRNA editing enzyme, catalytic polypeptide-like	Cytidine deaminase	Regulatory protein	High expression level	Mutations accumulation in DNA damage response and chromatin regulatory genes	Glaser et al., 2018 Jarvis et al., 2018 Shi et al., 2019
Chromatin modifying	KMT2D (MLL4)	Histone-lysine N-methyltransferase 2D	Histone H3 lysine 4 mono-methyltransferase	Essential for cell differenctiation, cell fate regulation, metabolism and tumor suppression	Loss of function or misregulation	Increase proliferative and migratory abilities	Sun et al., 2019 Ding et al., 2019 Hou et al., 2019
	KDM6A (UTX)	Lysine-specific demethylase 6A	Specific demethylase	Transcriptional regulation at promoters and enhancers	Mutations Loss of function	Promotes M2 macrophages polarization increasing cancer stem cells via cytokines	Hurst et al., 2017 Nickerson et al., 2014 Kaneko et al., 2018 Kobatake et al., 2020
	ARID1A	AT-rich interactive domain-containing protein 1A	Part of SWI/SNF family, Helicase and ATPase activities	Essential for transcriptional activation of genes normally repressed by chromatin	Mutations Loss of function	Involved in granting BC non-stem cells the capability of self-renewal.	Balbas-Martinez et al., 2013 Li et al., 2016 Yang et al., 2016
	BAP1	BRCA1 associated protein-1	Deubiquitinase	Involved in transcription-coupled nucleotide excision repair	Mutations Loss of function	BRCA pathway alteration	Nickerson et al., 2014 Lin et al., 2017 Tech et al., 2020
Signaling pathways	KRAS/HRAS/NRAS		GTP-binding proteins part of RTK/Ras pathway	Involved in transducing signal to regulate cell proliferation, survival and differenciation	Mutations Gain of function Loss of function	Hyperproliferative development disorders	Wu et al., 2015
	FGFR3	Fibroblast growth factor receptor 3	Part of the fibroblast growth factor receptor family	Involved in bone growth	MutationsGain of expression	Common feature of low-grade BC	The cancer genome atlas research network, 2014 Akanksha and Sandhya, 2019 Wu et al., 2015
	PIK3CA/Akt/mTOR	Phosphatidylinositol 3 kinase/Akt/mechanistic target of rapamycin	Intracellular signaling pathway	Involved in cell cycle regulation	Mutations Gain of function Loss of function	Involved in tumor growth and angiogenesis	Ching and Hansel, 2010
	TSC1/Hsp90	Tuberous sclerosis 1/Heat shock protein 90	Co-chaperone and chaperone proteins	TSC1 inhibits Hsp90 activity and regulates mTORC1 Hsp90 help for protein folding	Chr9 deletion Loss of function		Hornigold et al., 1999 Woodford et al., 2019 Knowles et al., 2003 Guo et al., 2013
	UPK1B	Uroplakin 1B	Cell surface protein mediating signal transduction	Involved in cell development, activation and growth	Upregulation	Promotes proliferation, invasion and migartion of cancerous cells	Wang et al., 2018
	IGFBP5	Insulin-like growth factor-binding protein 5	Transport protein	Transports IGF1 (Insulin like growth factor 1)	Overexpression		Liang et al., 2013 Neuzillet et al., 2017
	ERBB2	Erythroblastic oncogene-B2 receptor tyrosine kinase 2	Memeber of the human epidermal growth factor receptor family	promotes cell proliferation	Mutations Overexpression	Linked to development and progression of cancers, metastasis	Groenendijk et al., 2015 Yoshida et al., 2019
	NID2	Nidogen-2	Basal lamina protein	Plays a role during late embryonic development	Methylation status change	Important for BC development but mechanism not clear yet	Fantony et al., 2015 Fantony et al., 2017
Post-translational modification	MDM2	Murine double minute 2	E3 ubiquitin ligase	Responsible for p53 regulation	Mutations SNP at position 309		Gangwar and Devi Mittal, 2010 Xie et al., 2015 Horikawa et al., 2008
	CDK1	Cyclin-dependent kinase 1	Serine/threonine kinase	Key player in cell cycle regulation by allowing cell cycle progression	Overexpression	Promotes proliferation, invasion and self-renewal	Tian et al., 2018 Heo et al., 2020
	AURKA	Aurora kinase A	Serine/threonine kinase	Important for cell proliferation	Overexpression	Induces centrosome amplification, chromosome missegregation, aneuploidy and stimulates cell proliferation and invasion	Park et al., 2008 Mobley et al., 2017 Guo et al., 2018
Transcription factor	EOMES	Eomesodermin	Transcription factor	Important for development and immunity	Hypermethylation	Important for BC development but mechanism not clear yet	Reinert et al., 2012
	HOXA9	Homeobox protein Hox-9	Transcription factor	Involved in hematopoiesis and development	Hyper or Hypomethylation	Important for BC development but mechanism not clear yet	Kim et al., 2013 Kitchen et al., 2015
	POU4F2	POU domain, class 4, transcription factor 2	Transcription factor	Involved in maintaining visual system neurons	Hyper or Hypomethylation	Important for BC development but mechanism not clear yet	Reinert et al., 2012 Wang et al., 2015
	RUNX3	Runt-related transcription factor 3	Transcription factor	modulate the transcription of their target genes	Deleted or silenced due to hypermethylation or mutations		Kim et al., 2005 Wilff et al., 2008 Zhang et al., 2008 Yan et al., 2012
	TP53	Tumor protein 53	Transcription factor	Involved in cell cycle regulation, autophagy and apoptosis	Mutations		Pandith et al., 2010 Hoffman-Censits et al., 2019 Horikawa et al., 2008
	TERTp	Telomeras reverse transcriptase promoter	Promoter for a RNA-dependent telomerase	Lengthens telomeres	Mutations Loss of function		Batista et al., 2020 Nickerson et al., 2014
Growth factor	IGF2	Insulin-like growth factor 2	Hormone	Growth promoting hormone indispensable during gestation Involved in carbohydrates metabolism	Overexpression Hyper or Hypomethylation	Important for BC development but mechanism not clear yet	Byun et al., 2007 Chen et al., 2013
	ANXA10	Annexin 10	calcium-dependent phospholipid binding	Involved in cellular growth and in signal transduction	Lower expression	Important for BC development but mechanism not clear yet	Munksgaard et al., 2011
	NEGF2	neurite growth-promoting factor 2	Basic heparin-binding growth factor	Involved in cell proliferation, cell migration and survival	Overexpression	Involved in cell proliferation, cell migration and angiogenesis	Hunter et al, 2000 Sakamoto et al., 2012 Jones et al., 2014
Bladder cancer antigen	BLCA1/BLCA4	Bladder cancer-specific nuclear matrix protein	nuclear matrix protein	increases the levels of IL-1α, IL-8 and thrombomodulin	expressed only un BC	Promote tumor cell proliferation, survival and angiogenesis	Schawlb et al., 1993
	19A211 tumor-associated antigen: CEA	carcinoembryonic antigen	specific product of endodermally-derived neoplasms	cell adhesion	Absent form urothelial cells	Lower risk of tumor recurrence	Fradet et al., 1990 Bergeron et al., 1996 Têtu et al. 2005
	M344 and LDQ1053 tumor-associated antigens: MAUB	mucin antigen of the urinary bladder	high molecular weight glycoproteins	cell signaling, cell adhesions, differentiation of epithelial cells and immune response	aberrant regulation of mucin gene expression or aberrantglycosylation of the gene product	Aggressive biological behavior	Bergeron et al., 1996 Moniaux et al., 2001 Rachagani et al., 2009
Extracellular compartement	HA	Hyaluronic acid	Nonsulfated glycosaminoglycan	Component of tissue matrix and tissue fluids	Absent form urothelial cells		Lokeshwar et al., 2000
	hCFHrp	Human complement factor H-related protein	Member of the complement factor H family	Regulates factor H	Absent form urothelial cells	Promote growth and host immune system escaping	Kinders et al., 1998 Raitanen et al., 2001
	CD15s	Lewis X antigen/Sialyl LewisX (sLeX)/stage-specific embryonic antigen 1 (SSEA-1)	surface glycan/Blood group antigen	cell-to-cell recognition processes and fettilization	Absent form urothelial cells	Leukocyte adhesion deficiency	Mourant et al., 1946 Itzkowitz et al., 1986 Pode et al., 1998
	Fibrin/Fibrinogen	Fibrin/Fibrinogen	fibrous, non-globular protein	clotting of blood	overexpression		Schmetter et al., 1997
	EVs	Extracellular vesicles	lipid bilayer-delimited particles	proteins, nucleic acids, lipids, metabolites, and even organelles transport from the parent cell		Communication between tumor cells and stromal cells and local tumor progression, metastatic spread and the emergence of drug resistance	Lin et al., 2016 Silvers et al., 2017
Cell structure	CK	Cytokeratins	Intermediate filament proteins	Helps to resist to mechanical stress	Overexpression	Aberrant differentiation in the process of urothelial carcinogenesis and poor prognosis	Björklund et al., 1957 Lüning et al., 1980
Nuclear mitotic apparatus	NMP22	Nuclear matrix protein 22	nulcear protein	Important role in mitosis regulation	Overexpression	NMP22 is released from cells during apoptosis	Miyanaga et al., 1999 Bibbo et al., 2008 Balci et al., 2015
Apoptosis inhibitor	BIRC5	Baculovirus IAP repeat-containing protein 5 or Survivin	Member of the inhibitor of apoptosis protein (IAP)	Important only during fetal development	Overexpression	cell survive	Ambrosini et al., 1997 Li et al., 1998 Li et al., 1999 Altieri et al., 1999
Nuclear import/export	KPNA2	Karyopherin alpha 2	Member of the karyopherin family	Involved in cargo localization regulation	Overexpression	Increases proliferation, migration and invasion ability	Shi et al., 2020

## Data Availability

Not applicable.

## Supplementary Materials

The following are available online at https://www.mdpi.com/article/10.3390/cancers13071650/s1, Table S1: Performances of non-invasive tests.

## References

[1] Binder-Foucard F., Bossard N., Delafosse P., Belot A., Woronoff A.-S., Remontet L. (2014). Cancer Incidence and Mortality in France over the 1980–2012 Period: Solid Tumors. Rev. DÉpidémiol. Santé Publique.

[2] Leal J., Luengo-Fernandez R., Sullivan R., Witjes J.A. (2016). Economic Burden of Bladder Cancer Across the European Union. Eur. Urol..

[3] Farling K.B. (2017). Bladder Cancer: Risk Factors, Diagnosis, and Management. Nurse Pract..

[4] Amin M.B., Greene F.L., Edge S.B., Compton C.C., Gershenwald J.E., Brookland R.K., Meyer L., Gress D.M., Byrd D.R., Winchester D.P. (2017). The Eighth Edition AJCC Cancer Staging Manual: Continuing to Build a Bridge from a Population-Based to a More “Personalized” Approach to Cancer Staging. CA Cancer J. Clin..

[5] Griffiths T.R.L. (2013). Current Perspectives in Bladder Cancer Management. Int. J. Clin. Pract..

[6] Sharp V.J., Barnes K.T., Erickson B.A. (2013). Assessment of Asymptomatic Microscopic Hematuria in Adults. Am. Fam. Phys..

[7] Barkan G.A., Wojcik E.M., Nayar R., Savic-Prince S., Quek M.L., Kurtycz D.F.I., Rosenthal D.L. (2016). The Paris System for Reporting Urinary Cytology: The Quest to Develop a Standardized Terminology. Acta Cytol..

[8] Owens C.L., Vanden Bussche C.J., Burroughs F.H., Rosenthal D.L. (2013). A Review of Reporting Systems and Terminology for Urine Cytology. Cancer Cytopathol..

[9] Rouprêt M., Pignot G., Masson-Lecomte A., Compérat E., Audenet F., Roumiguié M., Houédé N., Larré S., Brunelle S., Xylinas E. (2020). Recommandations françaises du Comité de cancérologie de l’AFU—Actualisation 2020–2022: Tumeurs de la vessie. Prog. En Urol..

[10] Zhu C.-Z., Ting H.-N., Ng K.-H., Ong T.-A. (2019). A Review on the Accuracy of Bladder Cancer Detection Methods. J. Cancer.

[11] Malmström P.-U., Agrawal S., Bläckberg M., Boström P.J., Malavaud B., Zaak D., Hermann G.G. (2017). Non-Muscle-Invasive Bladder Cancer: A Vision for the Future. Scand. J. Urol..

[12] Pfister C., Roupret M., Neuzillet Y., Larré S., Pignot G., Quintens H., Houedé N., Compérat E., Colin P., Roy C. (2013). Recommandations en onco-urologie 2013 du CCAFU: Tumeurs de la vessie. Prog. Urol..

[13] Oeyen E., Hoekx L., De Wachter S., Baldewijns M., Ameye F., Mertens I. (2019). Bladder Cancer Diagnosis and Follow-Up: The Current Status and Possible Role of Extracellular Vesicles. Int. J. Mol. Sci..

[14] Sloan F.A., Yashkin A.P., Akushevich I., Inman B.A. (2020). The Cost to Medicare of Bladder Cancer Care. Eur. Urol. Oncol..

[15] Barratt J., Topham P. (2007). Urine Proteomics: The Present and Future of Measuring Urinary Protein Components in Disease. Can. Med. Assoc. J..

[16] Thomas C.E., Sexton W., Benson K., Sutphen R., Koomen J. (2010). Urine Collection and Processing for Protein Biomarker Discovery and Quantification. Cancer Epidemiol. Biomark. Prev..

[17] Raitanen M.-P., Kaasinen E., Rintala E., Hansson E., Nieminen P., Aine R., Tammela T.L.J., The Finn Bladder Group (2001). Prognostic Utility of Human Complement Factor H Related Protein Test (the BTA Stat^®^Test). Br. J. Cancer.

[18] Kinders R., Jones T., Root R., Bruce C., Murchison H., Corey M., Williams L., Enfield D., Hass G.M. (1998). Complement Factor H or a Related Protein Is a Marker for Transitional Cell Cancer of the Bladder. Clin. Cancer Res..

[19] Gutiérrez Baños J.L., Rodrigo M.d.H.R., Juárez F.M.A., García B.M. (2001). Usefulness of the BTA Stat Test for the Diagnosis of Bladder Cancer. Urology.

[20] Thomas L., Leyh H., Marberger M., Bombardieri E., Bassi P., Pagano F., Pansadoro V., Sternberg C.N., Boccon-Gibod L., Ravery V. (1999). Multicenter Trial of the Quantitative BTA TRAK Assay in the Detection of Bladder Cancer. Clin. Chem..

[21] Shariat S.F., Karam J.A., Lotan Y., Karakiewizc P.I. (2008). Critical Evaluation of Urinary Markers for Bladder Cancer Detection and Monitoring. Rev. Urol..

[22] Campos-Fernandes J.-L., Descotes F., André J., Perrin P., Devonec M., Ruffion A. (2007). Value of urinary markers in the diagnosis and follow-up of urothelial bladder tumours. Prog. Urol. J. Assoc. Fr. Urol. Soc. Fr. Urol..

[23] Budman L.I., Kassouf W., Steinberg J.R. (2008). Biomarkers for Detection and Surveillance of Bladder Cancer. Can. Urol. Assoc. J..

[24] Bibbo M., Kern W.H., Bibbo M., Wilbur D. (2008). Chapter 15—Urinary Tract. Comprehensive Cytopathology.

[25] Miyanaga N., Akaza H., Tsukamoto T., Ishikawa S., Noguchi R., Ohtani M., Kawabe K., Kubota Y., Fujita K., Obata K. (1999). Urinary Nuclear Matrix Protein 22 as a New Marker for the Screening of Urothelial Cancer in Patients with Microscopic Hematuria. Int. J. Urol..

[26] Balci M., Tuncel A., Guzel O., Aslan Y., Sezgin T., Bilgin O., Senel C., Atan A. (2015). Use of the Nuclear Matrix Protein 22 Bladder Chek Test^TM^ in the Diagnosis of Residual Urothelial Cancer before a Second Transurethral Resection of Bladder Cancer. Int. Urol. Nephrol..

[27] Gellhaus P.T., Genetic Aberrations in Bladder Cancer, Fluorescence in Situ Hybridization In Urine Tumor Markers in Bladder Cancer Diagnosis: Overview of Urine Tumor Markers; 2020. https://emedicine.medscape.com/article/1953022-overview.

[28] Mowatt G., Zhu S., Kilonzo M., Boachie C., Fraser C., Griffiths T.R.L., N’Dow J., Nabi G., Cook J., Vale L. (2010). Systematic Review of the Clinical Effectiveness and Cost-Effectiveness of Photodynamic Diagnosis and Urine Biomarkers (FISH, ImmunoCyt, NMP22) and Cytology for the Detection and Follow-up of Bladder Cancer. Health Technol. Assess..

[29] Moonen P.M.J., Kiemeney L.A.L.M., Witjes J.A. (2005). Urinary NMP22^®^ BladderChek^®^ Test in the Diagnosis of Superficial Bladder Cancer. Eur. Urol..

[30] Doğan C., Pelit E.S., Yıldırım A., Zemheri I.E., Çanakcı C., Başok E.K., Çaşkurlu T. (2013). The Value of the NMP22 Test for Superficial Bladder Cancer Diagnosis and Follow-Up. Turk. J. Urol..

[31] Kumar A., Kumar R., Gupta N.P. (2006). Comparison of NMP22 BladderChek Test and Urine Cytology for the Detection of Recurrent Bladder Cancer. Jpn. J. Clin. Oncol..

[32] Fradet Y., Larue H., Parent-Vaugeois C., Bergeron A., Dufour C., Boucher L., Bernier L. (1990). Monoclonal Antibody against a Tumor-Associated Sialoglycoprotein of Superficial Papillary Bladder Tumors and Cervical Condylomas. Int. J. Cancer.

[33] Bergeron A., LaRue H., Fradet Y. (1996). Identification of a Superficial Bladder Tumor-Associated Glycoform of the Carcinoembryonic Antigen by Monoclonal Antibody 19A211. Cancer Res..

[34] Têtu B., Tiguert R., Harel F., Fradet Y. (2005). ImmunoCyt/UCyt+^TM^ Improves the Sensitivity of Urine Cytology in Patients Followed for Urothelial Carcinoma. Mod. Pathol..

[35] Bergeron A., Champetier S., LaRue H., Fradet Y. (1996). MAUB Is a New Mucin Antigen Associated with Bladder Cancer. J. Biol. Chem..

[36] Bergeron A., LaRUE H., Fradet Y. (1997). Biochemical Analysis of a Bladder-Cancer-Associated Mucin: Structural Features and Epitope Characterization. Biochem. J..

[37] Allard P., Fradet Y., Têtu B., Bernard P. (1995). Tumor-Associated Antigens as Prognostic Factors for Recurrence in 382 Patients with Primary Transitional Cell Carcinoma of the Bladder. Clin. Cancer Res..

[38] Greene K.L., Berry A., Konety B.R. (2006). Diagnostic Utility of the ImmunoCyt/uCyt^+^ Test in Bladder Cancer. Rev. Urol..

[39] Piaton E., Daniel L., Verriele V., Dalifard I., Zimmermann U., Renaudin K., Gobet F., Caratero A., Desvaux D., Pouille Y. (2003). Improved Detection of Urothelial Carcinomas with Fluorescence Immunocytochemistry (UCyt+ Assay) and Urinary Cytology: Results of a French Prospective Multicenter Study. Lab. Investig..

[40] Schmitz-Dräger C., Bonberg N., Pesch B., Todenhöfer T., Sahin S., Behrens T., Brüning T., Schmitz-Dräger B.J. (2016). Replacing Cystoscopy by Urine Markers in the Follow-up of Patients with Low-Risk Non–Muscle-Invasive Bladder Cancer?—An International Bladder Cancer Network Project. Urol. Oncol. Semin. Orig. Investig..

[41] Kassouf W., Traboulsi S.L., Schmitz-Dräger B., Palou J., Witjes J.A., van Rhijn B.W.G., Grossman H.B., Kiemeney L.A., Goebell P.J., Kamat A.M. (2016). Follow-up in Non–Muscle-Invasive Bladder Cancer—International Bladder Cancer Network Recommendations. Urol. Oncol. Semin. Orig. Investig..

[42] Björklund B., Björklund V. (1957). Antigenicity of Pooled Human Malignant and Normal Tissues by Cyto-Immunological Technique: Presence of an Insoluble, Heat-Labile Tumor Antigen. Int. Arch. Allergy Immunol..

[43] Lüning B., Wiklund B., Redelius P., Björklund B. (1980). Biochemical Properties of Tissue Polypeptide Antigen. Biochim. Biophys. Acta BBA Protein Struct..

[44] Maulard-Durdux C., Toubert M.E., Hennequin C., Housset M. (1997). Serum Tissue Polypeptide Antigen in Bladder Cancer as a Tumor Marker: A Prospective Study. J. Clin. Oncol..

[45] Sánchez-Carbayo M., Urrutia M., Silva J.M., Romaní R., García J., Alférez F., González de Buitrago J.M., Navajo J.A. (2000). Urinary Tissue Polypeptide-Specific Antigen for the Diagnosis of Bladder Cancer. Urology.

[46] Ritter R., Hennenlotter J., Kühs U., Hofmann U., Aufderklamm S., Blutbacher P., Deja A., Hohneder A., Gerber V., Gakis G. (2014). Evaluation of a New Quantitative Point-of-Care Test Platform for Urine-Based Detection of Bladder Cancer. Urol. Oncol. Semin. Orig. Investig..

[47] Ecke T.H., Arndt C., Stephan C., Hallmann S., Lux O., Otto T., Ruttloff J., Gerullis H. (2015). Preliminary Results of a Multicentre Study of the UBC Rapid Test for Detection of Urinary Bladder Cancer. Anticancer Res..

[48] Ecke T.H., Weiß S., Stephan C., Hallmann S., Barski D., Otto T., Gerullis H. (2017). UBC^®^
*Rapid* Test for Detection of Carcinoma in Situ for Bladder Cancer. Tumor Biol..

[49] Pichler R., Tulchiner G., Fritz J., Schaefer G., Horninger W., Heidegger I. (2017). Urinary UBC Rapid and NMP22 Test for Bladder Cancer Surveillance in Comparison to Urinary Cytology: Results from a Prospective Single-Center Study. Int. J. Med. Sci..

[50] Mourant A.E. (1946). A ‘New’ Human Blood Group Antigen of Frequent Occurrence. Nature.

[51] Itzkowitz S.H., Yuan M., Fukushi Y., Palekar A., Phelps P.C., Shamsuddin A.M., Trump B.F., Hakomori S., Kim Y.S. (1986). Lewis^X^- and Sialylated Lewis^X^-Related Antigen Expression in Human Malignant and Nonmalignant Colonie Tissues. Cancer Res..

[52] Pode D., Golijanin D., Sherman Y., Lebensart P., Shapiro A. (1998). Immunostaining of Lewis X in cells from voided urine, cytopathology and ultrasound for noninvasive detection of bladder tumors. J. Urol..

[53] Planz B., Synek C., Deix T., Böcking A., Marberger M. (2001). Diagnosis of Bladder Cancer with Urinary Cytology, Immunocytology and DNA-Image-Cytometry. Anal. Cell. Pathol..

[54] Golijanin D., Sherman Y., Shapiro A., Pode D. (1995). Detection of Bladder Tumors by Immunostaininc of the Lewis x Antigen in Cells from Voided Urine. Urology.

[55] Ambrosini G., Adida C., Altieri D.C. (1997). A Novel Anti-Apoptosis Gene, Survivin, Expressed in Cancer and Lymphoma. Nat. Med..

[56] Li F., Ambrosini G., Chu E.Y., Plescia J., Tognin S., Marchisio P.C., Altieri D.C. (1998). Control of Apoptosis and Mitotic Spindle Checkpoint by Survivin. Nature.

[57] Li F., Ackermann E.J., Bennett C.F., Rothermel A.L., Plescia J., Tognin S., Villa A., Marchisio P.C., Altieri D.C. (1999). Pleiotropic Cell-Division Defects and Apoptosis Induced by Interference with Survivin Function. Nat. Cell Biol..

[58] Altieri D.C., Marchisio P.C., Marchisio C. (1999). Survivin Apoptosis: An Interloper between Cell Death and Cell Proliferation in Cancer. Lab. Investig..

[59] Reed J.C. (2001). The Survivin Saga Goes In Vivo. J. Clin. Investig..

[60] Satoh K., Kaneko K., Hirota M., Masamune A., Satoh A., Shimosegawa T. (2001). Expression of Survivin Is Correlated with Cancer Cell Apoptosis and Is Involved in the Development of Human Pancreatic Duct Cell Tumors. Cancer.

[61] Tanaka C., Uzawa K., Shibahara T., Yokoe H., Noma H., Tanzawa H. (2003). Expression of an Inhibitor of Apoptosis, Survivin, in Oral Carcinogenesis. J. Dent. Res..

[62] Dallaglio K., Marconi A., Pincelli C. (2012). Survivin: A Dual Player in Healthy and Diseased Skin. J. Investig. Dermatol..

[63] Eissa S., Swellam M., Shehata H., El-Khouly I.M., El-Zayat T., El-Ahmady O. (2010). Expression of *HYAL1* and Survivin RNA as Diagnostic Molecular Markers for Bladder Cancer. J. Urol..

[64] Wang H., Xi X., Kong X., Huang G., Ge G. (2004). The Expression and Significance of Survivin MRNA in Urinary Bladder Carcinomas. J. Cancer Res. Clin. Oncol..

[65] Smith S.D., Wheeler M.A., Plescia J., Colberg J.W., Weiss R.M., Altieri D.C. (2001). Urine Detection of Survivin and Diagnosis of Bladder Cancer. JAMA.

[66] Lokeshwar V.B., Block N.L. (2000). HA-HAase URINE TEST: A Sensitive and Specific Method for Detecting Bladder Cancer and Evaluating Its Grade. Urol. Clin. N. Am..

[67] Knudson W. (1996). Tumor-Associated Hyaluronan. Am. J. Pathol..

[68] Pham H.T., Block N.L., Lokeshwar V.B. (1997). Tumor-Derived Hyaluronidase: A Diagnostic Urine Marker for High-Grade Bladder Cancer. Cancer Res..

[69] Kramer M.W., Golshani R., Merseburger A.S., Knapp J., Garcia A., Hennenlotter J., Duncan R.C., Soloway M.S., Jorda M., Kuczyk M.A. (2010). HYAL-1 Hyaluronidase: A Potential Prognostic Indicator for Progression to Muscle Invasion and Recurrence in Bladder Cancer. Eur. Urol..

[70] Morera D.S., Hennig M.S., Talukder A., Lokeshwar S.D., Wang J., Garcia-Roig M., Ortiz N., Yates T.J., Lopez L.E., Kallifatidis G. (2017). Hyaluronic Acid Family in Bladder Cancer: Potential Prognostic Biomarkers and Therapeutic Targets. Br. J. Cancer.

[71] Van Tilborg A.A.G., Bangma C.H., Zwarthoff E.C. (2009). Bladder Cancer Biomarkers and Their Role in Surveillance and Screening. Int. J. Urol..

[72] Parker J., Spiess P.E. (2011). Current and Emerging Bladder Cancer Urinary Biomarkers. Sci. World J..

[73] Liang Z., Zhang Q., Wang C., Shi F., Cao H., Yu Y., Zhang M., Liu X. (2017). Hyaluronic Acid/ Hyaluronidase as Biomarkers for Bladder Cancer: A Diagnostic Meta-Analysis. Neoplasma.

[74] Konety B.R., Nguyen T.-S.T., Dhir R., Day R.S., Becich M.J., Stadler W.M., Getzenberg R.H. (2000). Detection of Bladder Cancer Using a Novel Nuclear Matrix Protein, BLCA-4. Clin. Cancer Res..

[75] Getzenberg R.H., Konety B.R., Oeler T.A., Quigley M.M., Hakam A., Becich M.J., Bahnson R.R. (1996). Bladder Cancer-Associated Nuclear Matrix Proteins. Cancer Res..

[76] Myers-Irvin J.M., Landsit D., Getzenberg R.H. (2005). Use of the novel marker blca-1 for the detection of bladder cancer. J. Urol..

[77] Santoni M., Catanzariti F., Minardi D., Burattini L., Nabissi M., Muzzonigro G., Cascinu S., Santoni G. (2012). Pathogenic and Diagnostic Potential of BLCA-1 and BLCA-4 Nuclear Proteins in Urothelial Cell Carcinoma of Human Bladder. Adv. Urol..

[78] Lotan Y., Roehrborn C.G. (2003). Sensitivity and Specificity of Commonly Available Bladder Tumor Markers versus Cytology: Results of a Comprehensive Literature Review and Meta-Analyses. Urology.

[79] Cai Q., Wu Y., Guo Z., Gong R., Tang Y., Yang K., Li X., Guo X., Niu Y., Zhao Y. (2015). Urine BLCA-4 Exerts Potential Role in Detecting Patients with Bladder Cancers: A Pooled Analysis of Individual Studies. Oncotarget.

[80] Wang S., Xia T., Zhang Z., Kong X., Zeng L., Mi P., Xue Z. (2000). Expression of VEGF and Tumor Angiogenesis in Bladder Cancer. Zhonghua Wai Ke Za Zhi.

[81] Schmetter B.S., Habicht K.K., Lamm D.L., Morales A., Bander N.H., Grossman H.B., Hanna M.G.J., Silberman S.R., Butman B.T. (1997). A Multicenter Trial Evaluation of the Fibrin/Fibrinogen Degradation Products Test for Detection and Monitoring of Bladder Cancer. J. Urol..

[82] Topsakal M., Karadeniz T., Anaç M., Dönmezer S., Besisik A. (2001). Assessment of Fibrin–Fibrinogen Degradation Products (Accu–Dx) Test in Bladder Cancer Patients. Eur. Urol..

[83] Wajsman Z., Williams P.D., Greco J., Murphy G.P. (1978). Further Study of Fibrinogen Degradation Products in Bladder Cancer Detection. Urology.

[84] Label INCa-HAS—Surveillance Médico-Professionnelle des Travailleurs Exposés ou Ayant Eté Exposés à des Agents Cancérogènes Chimiques: Application aux Cancérogènes Pour la Vessie. https://www.has-sante.fr/jcms/c_1246108/fr/label-inca-has-surveillance-medico-professionnelle-des-travailleurs-exposes-ou-ayant-ete-exposes-a-des-agents-cancerogenes-chimiques-application-aux-cancerogenes-pour-la-vessie.

[85] Silvers C.R., Miyamoto H., Messing E.M., Netto G.J., Lee Y.-F. (2017). Characterization of Urinary Extracellular Vesicle Proteins in Muscle-Invasive Bladder Cancer. Oncotarget.

[86] Lin S.-Y., Chang C.-H., Wu H.-C., Lin C.-C., Chang K.-P., Yang C.-R., Huang C.-P., Hsu W.-H., Chang C.-T., Chen C.-J. (2016). Proteome Profiling of Urinary Exosomes Identifies Alpha 1-Antitrypsin and H2B1K as Diagnostic and Prognostic Biomarkers for Urothelial Carcinoma. Sci. Rep..

[87] Silvers C.R., Liu Y.-R., Wu C.-H., Miyamoto H., Messing E.M., Lee Y.-F. (2016). Identification of Extracellular Vesicle-Borne Periostin as a Feature of Muscle-Invasive Bladder Cancer. Oncotarget.

[88] Hunter T. (2000). Signaling—2000 and Beyond. Cell.

[89] Sakamoto K., Kadomatsu K. (2012). Midkine in the Pathology of Cancer, Neural Disease, and Inflammation. Pathol. Int..

[90] Jones D.R. (2014). Measuring Midkine: The Utility of Midkine as a Biomarker in Cancer and Other Diseases. Br. J. Pharmacol..

[91] Ikematsu S., Okamoto K., Yoshida Y., Oda M., Sugano-Nagano H., Ashida K., Kumai H., Kadomatsu K., Muramatsu H., Muramatsu T. (2003). High Levels of Urinary Midkine in Various Cancer Patients. Biochem. Biophys. Res. Commun..

[92] Shimwell N.J., Bryan R.T., Wei W., James N.D., Cheng K.K., Zeegers M.P., Johnson P.J., Martin A., Ward D.G. (2013). Combined Proteome and Transcriptome Analyses for the Discovery of Urinary Biomarkers for Urothelial Carcinoma. Br. J. Cancer.

[93] Soukup V., Kalousová M., Capoun O., Sobotka R., Breyl Z., Pešl M., Zima T., Hanuš T. (2015). Panel of Urinary Diagnostic Markers for Non-Invasive Detection of Primary and Recurrent Urothelial Urinary Bladder Carcinoma. Urol. Int..

[94] Vu Van D., Heberling U., Wirth M.P., Fuessel S. (2016). Validation of the Diagnostic Utility of Urinary Midkine for the Detection of Bladder Cancer. Oncol. Lett..

[95] Luo Y., She D.-L., Xiong H., Yang L., Fu S.-J. (2015). Diagnostic Value of Liquid-Based Cytology in Urothelial Carcinoma Diagnosis: A Systematic Review and Meta-Analysis. PLoS ONE.

[96] Kapoor K., Datta C., Pal D.K. (2019). Is Liquid-Based Cytology an Alternative to Conventional Cytology for Detection of Malignant Cells in Urine of Bladder Cancer? Eastern Indian Prospective Observational Study. Turk. J. Urol..

[97] Lee H., Kim K., Woo J., Park J., Kim H., Lee K.E., Kim H., Kim Y., Moon K.C., Kim J.Y. (2018). Quantitative Proteomic Analysis Identifies AHNAK (Neuroblast Differentiation-Associated Protein AHNAK) as a Novel Candidate Biomarker for Bladder Urothelial Carcinoma Diagnosis by Liquid-Based Cytology. Mol. Cell. Proteom..

[98] Sokolova I.A., Halling K.C., Jenkins R.B., Burkhardt H.M., Meyer R.G., Seelig S.A., King W. (2000). The Development of a Multitarget, Multicolor Fluorescence in Situ Hybridization Assay for the Detection of Urothelial Carcinoma in Urine. J. Mol. Diagn. JMD.

[99] van Valenberg F.J.P., Strauss-Ayali D., Agmon-Gerstein Y., Friedman A., Arentsen H.C., Schaafsma H.E., Witjes J.A., Oosterwijk E. (2018). Assessment of the Efficacy of Repeated Instillations of Mitomycin C Mixed with a Thermosensitive Hydrogel in an Orthotopic Rat Bladder Cancer Model. Ther. Adv. Urol..

[100] Yoder B.J., Skacel M., Hedgepeth R., Babineau D., Ulchaker J.C., Liou L.S., Brainard J.A., Biscotti C.V., Jones J.S., Tubbs R.R. (2007). Reflex UroVysion Testing of Bladder Cancer Surveillance Patients with Equivocal or Negative Urine Cytology: A Prospective Study with Focus on the Natural History of Anticipatory Positive Findings. Am. J. Clin. Pathol..

[101] Kojima T., Nishiyama H., Ozono S., Hinotsu S., Keino N., Yamaguchi A., Sakai H., Enomoto Y., Horie S., Fujimoto K. (2018). Clinical Evaluation of Two Consecutive UroVysion Fluorescence in Situ Hybridization Tests to Detect Intravesical Recurrence of Bladder Cancer: A Prospective Blinded Comparative Study in Japan. Int. J. Clin. Oncol..

[102] Holyoake A., O’Sullivan P., Pollock R., Best T., Watanabe J., Kajita Y., Matsui Y., Ito M., Nishiyama H., Kerr N. (2008). Development of a Multiplex RNA Urine Test for the Detection and Stratification of Transitional Cell Carcinoma of the Bladder. Clin. Cancer Res..

[103] O’Sullivan P., Sharples K., Dalphin M., Davidson P., Gilling P., Cambridge L., Harvey J., Toro T., Giles N., Luxmanan C. (2012). A Multigene Urine Test for the Detection and Stratification of Bladder Cancer in Patients Presenting with Hematuria. J. Urol..

[104] Kavalieris L., O’Sullivan P.J., Suttie J.M., Pownall B.K., Gilling P.J., Chemasle C., Darling D.G. (2015). A Segregation Index Combining Phenotypic (Clinical Characteristics) and Genotypic (Gene Expression) Biomarkers from a Urine Sample to Triage out Patients Presenting with Hematuria Who Have a Low Probability of Urothelial Carcinoma. BMC Urol..

[105] Darling D., Luxmanan C., O’Sullivan P., Lough T., Suttie J. (2017). Clinical Utility of Cxbladder for the Diagnosis of Urothelial Carcinoma. Adv. Ther..

[106] Lough T., Luo Q., O’Sullivan P., Chemaslé C., Stotzer M., Suttie J., Darling D. (2018). Clinical Utility of Cxbladder Monitor for Patients with a History of Urothelial Carcinoma: A Physician-Patient Real-World Clinical Data Analysis. Oncol. Ther..

[107] Dudley J.C., Schroers-Martin J., Lazzareschi D.V., Shi W.Y., Chen S.B., Esfahani M.S., Trivedi D., Chabon J.J., Chaudhuri A.A., Stehr H. (2019). Detection and Surveillance of Bladder Cancer Using Urine Tumor DNA. Cancer Discov..

[108] Springer S.U., Chen C.-H., Rodriguez Pena M.D.C., Li L., Douville C., Wang Y., Cohen J.D., Taheri D., Silliman N., Schaefer J. (2018). Non-Invasive Detection of Urothelial Cancer through the Analysis of Driver Gene Mutations and Aneuploidy. eLife.

[109] Eich M.-L., Rodriguez Pena M.D.C., Springer S.U., Taheri D., Tregnago A.C., Salles D.C., Bezerra S.M., Cunha I.W., Fujita K., Ertoy D. (2019). Incidence and Distribution of UroSEEK Gene Panel in a Multi-Institutional Cohort of Bladder Urothelial Carcinoma. Mod. Pathol..

[110] de Jong J.J., Liu Y., Robertson A.G., Seiler R., Groeneveld C.S., van der Heijden M.S., Wright J.L., Douglas J., Dall’Era M., Crabb S.J. (2019). Long Non-Coding RNAs Identify a Subset of Luminal Muscle-Invasive Bladder Cancer Patients with Favorable Prognosis. Genome Med..

[111] van Kessel K.E.M., Beukers W., Lurkin I., Ziel-van der Made A., van der Keur K.A., Boormans J.L., Dyrskjøt L., Márquez M., Ørntoft T.F., Real F.X. (2017). Validation of a DNA Methylation-Mutation Urine Assay to Select Patients with Hematuria for Cystoscopy. J. Urol..

[112] Park H.-S., Park W.S., Bondaruk J., Tanaka N., Katayama H., Lee S., Spiess P.E., Steinberg J.R., Wang Z., Katz R.L. (2008). Quantitation of Aurora Kinase A Gene Copy Number in Urine Sediments and Bladder Cancer Detection. J. Natl. Cancer Inst..

[113] Mobley A., Zhang S., Bondaruk J., Wang Y., Majewski T., Caraway N.P., Huang L., Shoshan E., Velazquez-Torres G., Nitti G. (2017). Aurora Kinase A Is a Biomarker for Bladder Cancer Detection and Contributes to Its Aggressive Behavior. Sci. Rep..

[114] Burgess E.F., Livasy C., Trufan S., Hartman A., Guerreri R., Naso C., Clark P.E., Grigg C., Symanowski J., Raghavan D. (2019). High Aurora Kinase Expression Identifies Patients with Muscle-Invasive Bladder Cancer Who Have Poor Survival after Neoadjuvant Chemotherapy. Urol. Oncol..

[115] Wang L., Shi J., Huang Y., Liu S., Zhang J., Ding H., Yang J., Chen Z. (2019). A Six-Gene Prognostic Model Predicts Overall Survival in Bladder Cancer Patients. Cancer Cell Int..

[116] Reinert T., Borre M., Christiansen A., Hermann G.G., Ørntoft T.F., Dyrskjøt L. (2012). Diagnosis of Bladder Cancer Recurrence Based on Urinary Levels of EOMES, HOXA9, POU4F2, TWIST1, VIM, and ZNF154 Hypermethylation. PLoS ONE.

[117] Maldonado L., Brait M., Michailidi C., Munari E., Driscoll T., Schultz L., Bivalacqua T., Schoenberg M., Sidransky D., Netto G.J. (2014). An Epigenetic Marker Panel for Recurrence Risk Prediction of Low Grade Papillary Urothelial Cell Carcinoma (LGPUCC) and Its Potential Use for Surveillance after Transurethral Resection Using Urine. Oncotarget.

[118] Leal A., Sidransky D., Brait M. (2020). Tissue and Cell-Free DNA-Based Epigenomic Approaches for Cancer Detection. Clin. Chem..

[119] D’Andrea D., Soria F., Zehetmayer S., Gust K.M., Korn S., Witjes J.A., Shariat S.F. (2019). Diagnostic Accuracy, Clinical Utility and Influence on Decision-Making of a Methylation Urine Biomarker Test in the Surveillance of Non-Muscle-Invasive Bladder Cancer. BJU Int..

[120] Hermanns T., Savio A.J., Olkhov-Mitsel E., Mari A., Wettstein M.S., Saba K., Bhindi B., Kuk C., Poyet C., Wild P.J. (2020). A Noninvasive Urine-Based Methylation Biomarker Panel to Detect Bladder Cancer and Discriminate Cancer Grade. Urol. Oncol..

[121] Steinbach D., Kaufmann M., Hippe J., Gajda M., Grimm M.-O. (2020). High Detection Rate for Non-Muscle-Invasive Bladder Cancer Using an Approved DNA Methylation Signature Test. Clin. Genitourin. Cancer.

[122] Zhang N., Chen S., Wu L., Wu Y., Jiang G., Shao J., Chen L., Sun J., Na R., Wang X. (2019). Identification of Cancer-Specific Methylation of Gene Combination for the Diagnosis of Bladder Cancer. J. Cancer.

[123] Batista R., Vinagre J., Prazeres H., Sampaio C., Peralta P., Conceição P., Sismeiro A., Leão R., Gomes A., Furriel F. (2019). Validation of a Novel, Sensitive, and Specific Urine-Based Test for Recurrence Surveillance of Patients with Non-Muscle-Invasive Bladder Cancer in a Comprehensive Multicenter Study. Front. Genet..

[124] Birkó Z., Nagy B., Klekner Á., Virga J. (2020). Novel Molecular Markers in Glioblastoma-Benefits of Liquid Biopsy. Int. J. Mol. Sci..

[125] Pisapia P., Malapelle U., Troncone G. (2019). Liquid Biopsy and Lung Cancer. Acta Cytol..

[126] Alimirzaie S., Bagherzadeh M., Akbari M.R. (2019). Liquid Biopsy in Breast Cancer: A Comprehensive Review. Clin. Genet..

[127] Wang H., Men C.-P. (2015). Correlation of Increased Expression of MicroRNA-155 in Bladder Cancer and Prognosis. Lab. Med..

[128] Yin X.-H., Jin Y.-H., Cao Y., Wong Y., Weng H., Sun C., Deng J.-H., Zeng X.-T. (2019). Development of a 21-MiRNA Signature Associated With the Prognosis of Patients With Bladder Cancer. Front. Oncol..

[129] Bi J., Liu H., Dong W., Xie W., He Q., Cai Z., Huang J., Lin T. (2019). Circular RNA Circ-ZKSCAN1 Inhibits Bladder Cancer Progression through MiR-1178-3p/P21 Axis and Acts as a Prognostic Factor of Recurrence. Mol. Cancer.

[130] Kutwin P., Konecki T., Jabłonowski Z. (2018). Urine MiRNA as a Potential Biomarker for Bladder Cancer Detection—A Meta-Analysis. Cent. Eur. J. Urol..

