# Supplementary material for: Noninvasive Urine-Based Tests to Diagnose or Detect Recurrence of Bladder Cancer
Source: Cancers (Basel). 2021 Apr 1;13(7):1650. doi: 10.3390/cancers13071650 (PMC8036846; doi:10.3390/cancers13071650)
Supplement: Supplementary file 1 [file cancers-13-01650-s001.pdf]

Table 1. Performances of non-invasive tests.

Test	Specificity	Sensitivity	Cohort	Publication
BTA-TRAK-BTA-STAT	68-72%	57-83%	from 40 to 163 patients (total of 1874)	[29-21, 23]
Alere NMP22-NMP22 BladderChek	79%	68%-65%	Total of 22 260 patients	[28-30]
Immunocyt-uCyt+	62%-84,2%	38,5%-92,1%	Total of 4 199 patients	[30,38-39]
UBC Rapid test	77,3%-81,8%	61,3%-64,5%	From to 31 to 452 (total of 773)	[46-49]
Lewis X antigen	85%	75%	From 101 to 260 (total of 361)	[52, 54]
Survivin	80%-90%	100%	From 30 to 46 (total of 188)	[64, 65]
HA-Haase test	82,50%	90,80%	From 51 to 504 (total of 1613)	[66, 73]
BLCA-1	87%	80%	71 patients	[76]
BLCA-4	97%	93%	From 76 to 253 patients (total of 1372)	[78, 79]
Accu-Dx FDP	68%-86%	52%-83%	From 56 to 97 patients (total of 153)	[82, 83]
Tumor-secreted extracellular vesicles	87,60%	62,70%	From 6 to 191 patients (total of 197)	[85, 86]
Urinary midkine protein	77,90%	69,70%	162 patients	[94]
Urovysion	80%-92%	74%-84%	From 179 to 400 patients	[98-101]
Xpert bladder cancer monitor	91%	84%	140 patients	[49]
CxBladder detect	85%-90%	47%-100%	From 152 to 485 patients	[102-105]
uCAPP-Seq		77%-100%	185 patients	[107]
UroSEEK	80%-93%	83%-95%	From 537 to 583 patients	[108-110]
Assure Mdx	86%	93%	200 patients	[111]
AURKA	80%-97%	80%-87%	From 248 to 487 patients	[112-115]
DNA methylation	94%-100%	82%-94%	From 36 to 440 patients	[116-122]
Uromonitor	93%	74%	185 patients	[123]
